# Developing a 10-Layer Retinal Segmentation for MacTel Using Semi-Supervised Learning

**DOI:** 10.1167/tvst.13.11.2

**Published:** 2024-11-05

**Authors:** Aayush Verma, Simone Tzaridis, Marian Blazes, Martin Friedlander, Aaron Y. Lee, Yue Wu

**Affiliations:** 1 Department of Ophthalmology, University of Washington, Seattle, WA, USA; 2 Roger and Angie Karalis Johnson Retina Center, Seattle, WA, USA; 3 The Scripps Research Institute, La Jolla, CA, USA; 4 The Lowy Medical Research Institute, La Jolla, CA, USA

**Keywords:** macular telangiectasia type 2, optical coherence tomography, deep learning, semisupervised learning, retinal layer segmentation

## Abstract

**Purpose:**

Automated segmentation software in optical coherence tomography (OCT) devices is usually developed for and primarily tested on common diseases. Therefore segmentation accuracy of automated software can be limited in eyes with rare pathologies.

**Methods:**

We sought to develop a semisupervised deep learning segmentation model that segments 10 retinal layers and four retinal features in eyes with Macular Telangiectasia Type II (MacTel) using a small labeled dataset by leveraging unlabeled images. We compared our model against popular supervised and semisupervised models, as well as conducted ablation studies on the model itself.

**Results:**

Our model significantly outperformed all other models in terms of intersection over union on the 10 retinal layers and two retinal features in the test dataset. For the remaining two features, the pre-retinal space above the internal limiting membrane and the background below the retinal pigment epithelium, all of the models performed similarly. Furthermore, we showed that using more unlabeled images improved the performance of our semisupervised model.

**Conclusions:**

Our model improves segmentation performance over supervised models by leveraging unlabeled data. This approach has the potential to improve segmentation performance for other diseases, where labeled data is limited but unlabeled data abundant.

**Translational Relevance:**

Improving automated segmentation of MacTel pathology on OCT imaging by leveraging unlabeled data may enable more accurate assessment of disease progression, and this approach may be useful for improving feature identification and location on OCT in other rare diseases as well.

## Introduction

The adoption of deep learning has transformed medical image analysis, with meaningful clinical applications such as segmentation of anatomic features, prediction of clinical outcomes, and suggestions of possible treatment approaches.^1^^–^^3^ In particular, the combination of optical coherence tomography (OCT),^4^ which has become widely used throughout ophthalmology to capture high-resolution details of the retinal microstructure, and deep learning segmentation models enables accurate detection, location, and segmentation of ocular features. The accurate segmentation of features such as retinal layers, cysts, drusen, and intraretinal fluid can be crucial for early disease diagnosis and subsequent treatment. Several deep learning frameworks have been successfully applied to automated segmentation of the retinal layers.^5^^–^^8^

One limitation of the current generation of deep learning models is that they are trained in a supervised manner, where a loss objective is minimized with respect to a labeled training dataset that consists of pairs of input images and their corresponding target labels or segmentation masks. This supervised approach has two main limitations. First, acquiring labeled data is time-consuming and expensive, especially for segmentation labels, which require human experts to manually trace target features pixel by pixel.^9^ Second and more importantly, it does not generalize to cases that were not included in the training data and from the same devices. This second limitation can be particularly critical in rare diseases displaying specific morphological characteristics. The accuracy of automatic segmentation of OCT scans from manufacturers, such as Heidelberg Engineering, decreases on disease-related changes. For example, when cysts and collapsed layers appear on OCT scans, the automatic segmentation models will fail to accurately segment the retinal layers. Two main traditional approaches exist to make supervised models more robust to rare diseases and their unique features. The first approach transforms the data so that less common features are boosted and better represented by adding augmented versions of these examples or by tuning the loss function to overweight rarer cases.^10^ The second approach acquires additional labeled data that is then incorporated into the training data, and the whole model is then retrained from scratch or through transfer learning.^11^ However, acquiring labeled data is time-consuming and even challenging to obtain in rare diseases.

Recently, self-supervised and semisupervised deep learning algorithms have been proposed to improve segmentation performance. This enables more data to be leveraged without having to manually label any additional images. Self-supervised approaches include Sedai et al,^12^ where an autoencoder is used to learn features about fundus images and then transfer learns from these features to a segmentation decoder network. Semi-supervised learning approaches include Chen et al.,^13^ where the labeled data is augmented with pseudo-labels iteratively. Although this is an interesting first attempt at semisupervised learning for ophthalmic images, this approach is susceptible to divergent pseudo-labels that can crowd out real labels. Sedai et al.^14^ overcomes this pseudo-label divergence problem by training a pair of student-teacher models, where the teacher model provides pseudo-label targets for the student model.

Self-supervised and semisupervised approaches can be particularly useful for rare diseases, such as Macular telangiectasia type 2 (MacTel), with few labeled training examples, but more unlabeled images. MacTel is a primarily neurodegenerative retinal disease with additional vascular alterations.^10^ The diagnosis of MacTel is based on characteristic findings on fundoscopy and multimodal retinal imaging, including OCT. On OCT, morphological changes include hyporeflective cavities within inner and outer retinal layers (“cysts”) without thickening of the retina, a disruption of outer retinal layers, and hyperreflective outer retinal changes. With disease progression, atrophic changes within the outer retina and a subsequent collapse of inner retinal layers may be observed.

In this study, we adopt the semisupervised approach using cross-pseudo supervision (CPS)^15^ and incorporate additional, unlabeled data to improve layer and feature segmentation. We systematically conducted ablation studies on our semisupervised approach and compared it with the popular semisupervised model, Mean Teacher,^16^ and a multitude of different supervised learning methods, as well as the manufacturer's automatic segmenter.

## Methods

### Data

This study was conducted in accordance with the Declaration of Helsinki. Imaging data was obtained from participants in the MacTel Project. The MacTel Project is a collaboration of 49 clinical sites in seven countries. Each participant in the MacTel Project was 18 years of age or older and enrolled into the Natural History Study after a diagnosis of MacTel was confirmed on clinical examination at the study sites. Diagnoses were based on stereoscopic color fundus photographs, OCT, fluorescein angiography and fundus autofluorescence images that were graded by the Reading Center at Moorfields Eye Hospital, London, UK. Each participating clinical site obtained approval from their institutional review board or independent ethics committee for the protocol and each participant provided written informed consent.

We collected a total of 592 Heidelberg Spectralis OCT screenshots from 149 patients and the corresponding automatically segmented layer boundary lines generated by Heidelberg's internal software. In addition, an unsupervised dataset was created from 4436 unannotated OCT scans from 200 patients.

No distinction was made between the eyes of the patients. Within the dataset, some patients have both eyes included with varying degrees of MacTel whereas others only have one eye.

### Data Processing

Spectral Domain-OCT scans were performed following a predefined imaging protocol, capturing volume scans of 15° × 10° in high-resolution mode, with a minimum of 97 scans (Spectralis; Heidelberg Engineering, Heidelberg, Germany), centered on the fovea. Before creating and training each of the models, data was extracted and preprocessed. With the Heidelberg Software, segmentation lines cannot be exported with raw OCT files. This is why window captures were used to extract segmentation data from the Heidelberg OCT images. The dataset included screenshots of the raw OCT images and screenshots of the same OCT scans with the automatic Heidelberg annotations. Screenshots of single OCT B-scans were taken using OBS Studio (version 30.0.0) following a predefined protocol. Using the Segmentation Editor of the Heidelberg Eye Explorer (Heidelberg Engineering), the automatic segmentation of a B-scan was extracted with OBS Studio. Each raw OCT screenshot image underwent a two-step preprocessing protocol: (1) isolation of the OCT scan from the screenshot image and (2) extraction of a 512 × 512 crop centered around the fovea. To create ground truth segmentation masks from the annotated OCT screenshots, each annotation screenshot underwent the same two-step preprocessing protocol as the raw OCT screenshots, as well as an additional two preprocessing steps: (3) The automatically generated Heidelberg layer boundary lines were manually corrected by a clinician as needed, and the corrected segmentation lines were extracted as well. (4) Then, to create ground truth masks for deep learning models, the pixels in the area between the boundary lines were assigned the class of the line that preceded them (the pixels between layer 1 and layer 2 are assigned class 1, and so on).

**Figure 1.**
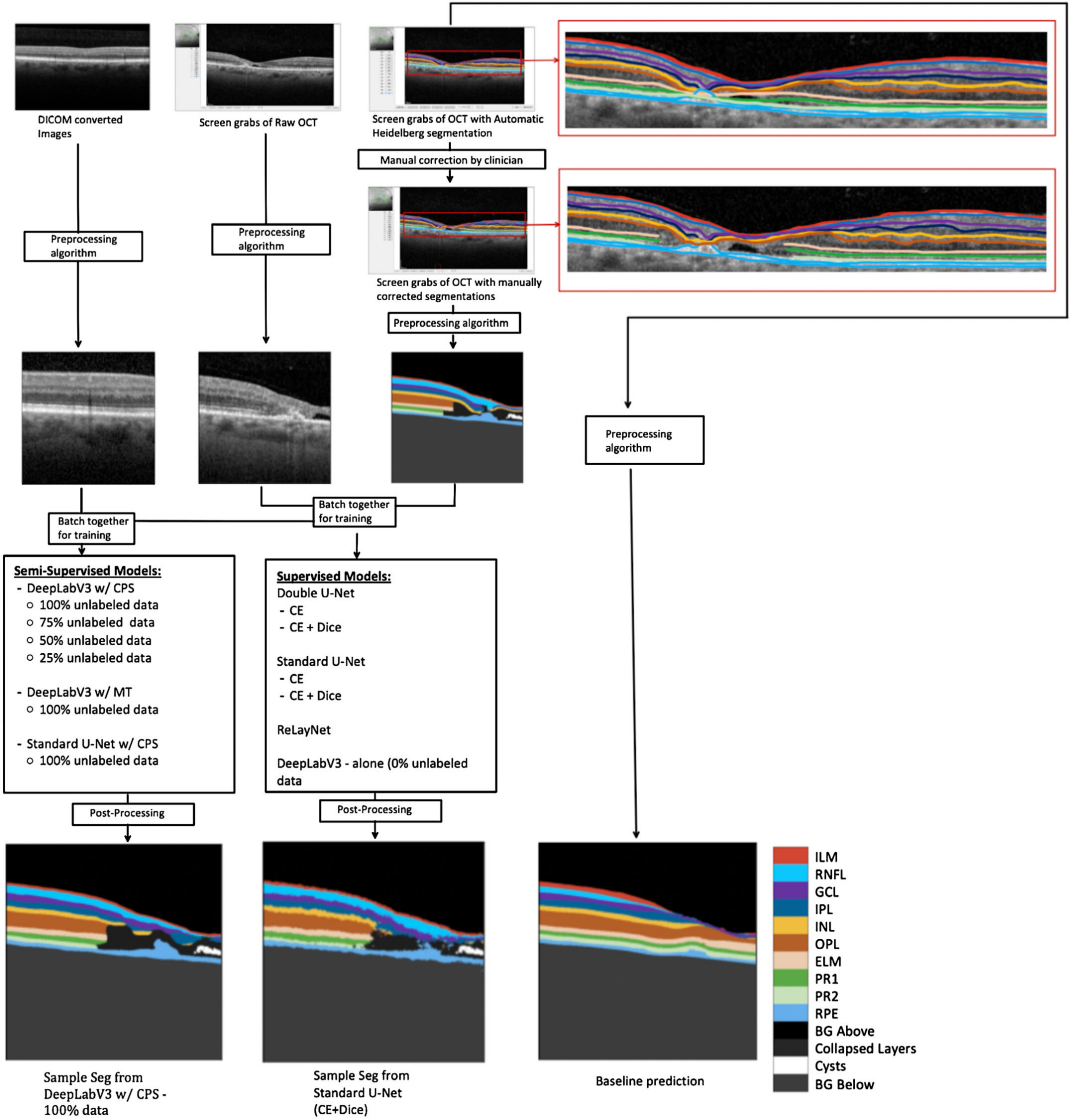
Schema showing the pre-processing and training protocol.

This process was repeated two to three times for each OCT screenshot and its corresponding annotated screenshot, depending on the size of the underlying B-scan, which ranged from 512px*496px to 784px*496px to 1024*496px, to extract from left to right tiled 512 × 512 pixels image patches and the corresponding annotated masks. Figure 1 illustrates this process. More details on the mask creation is given in the Supplementary Appendix and Supplementary Figure S1. There was no overlapping between any of the crops.

In total, 1707 pairs of input images and ground truth masks (each of dimension 512 × 512) were extracted from the 592 pairs of raw and annotated OCT screenshots collected, and an additional 13308 unlabeled input images were extracted from 4436 unannotated OCT scans using the same preprocessing protocol described above. Of the labeled input image and ground truth pairs, 292 of them were from OCT scans of eyes with varying levels of MacTel whereas the remaining were of eyes with no pathology. The scans were split 80:10:10 at the patient level to create a training, validation, and test set, and each training image was normalized with its individual mean and standard deviation before being passed through the different models as input data. The test set was composed of 74 diseased and 140 nonpathological images.

### Deep Learning Models

We sought to develop a method to reliably segment ten retinal layers and four additional features. The 10 retinal layers were internal limiting membrane (ILM), retinal nerve fiber layer, ganglion cell layer, inner plexiform layer, inner nuclear layer, outer plexiform layer, external limiting membrane, photoreceptor 1, photoreceptor 2, and retinal pigment epithelium (RPE). The four additional features were (i) collapsed layers, (ii) cysts, (iii) preretinal space, and (iv) background below retina. After data creation, we trained, tuned, and systematically compared 14 deep-learning segmentation models, six supervised and eight semisupervised, on segmenting the 14 different retinal features.

### Supervised Models

The supervised models were trained with labeled data only. The first baseline supervised model was the popular U-Net (referred to hereon as Standard UNet).^17^ A weighted cross entropy was used for training so that more emphasis is placed on the thinner layers such as the photoreceptor and collapsed layers. The second baseline model consisted of two chained U-Nets (referred to here on as Double UNet).^18^ To generate a prediction, the input image is first passed through the first chained UNet, which produces an interim prediction, as shown in Supplementary Figure S2. This interim prediction is concatenated with the initial input image as the input to the second chained, UNet, which generates an output (Supplementary Fig. S2). Both UNet and Double Net were also trained with the CrossEntropy + Dice loss, which have been used to improve segmentation results on OCTs. Another baseline supervised model trained was ReLayNet,^19^ which has a similar architecture to the UNet, but with a slightly different number of encoding and decoding layers. The current state-of-the-art supervised OCT segmentation model, DConnNet,^20^ was also trained. Finally, a state-of-the-art supervised ImageNet^21^ segmentation model, DeepLabV3,^22^ was trained. Both DConnNet and DeepLabV3 were used as a reference for ablation analysis of our supervised approach.

### Semisupervised Models

**Figure 2.**
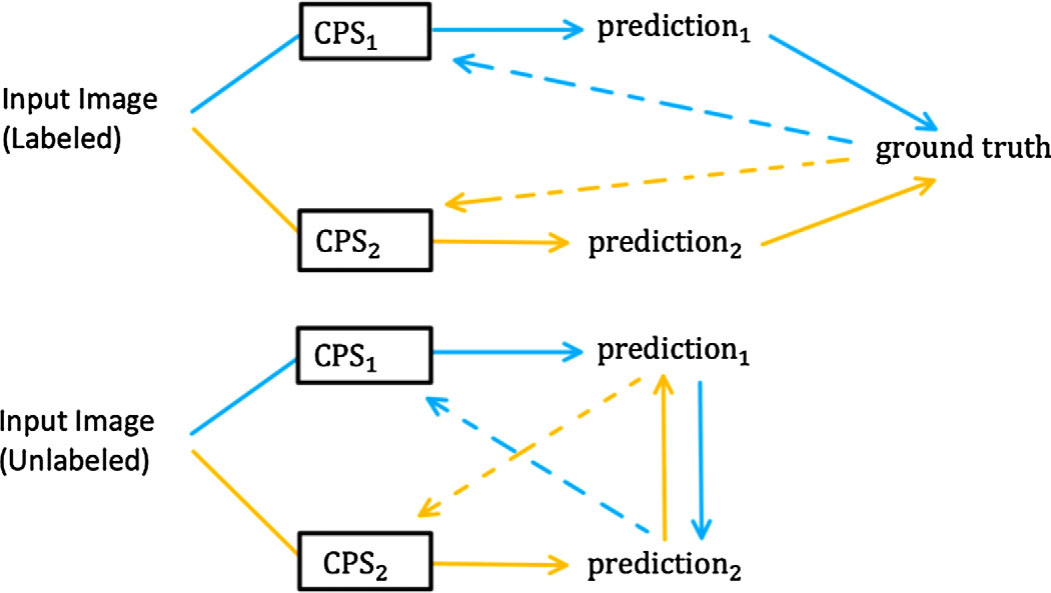
Schematic for DeepLabV3 w/CPS. When a labeled input image is passed through, the predictions are compared against the ground truth and back propagated as usual for both models. When an unlabeled image is passed through, the prediction from the first component model is used as the ground truth for the second component model and vice versa. Standard UNet w/CPs and DConnNet w/CPS are trained in a similar fashion.

Our semisupervised deep learning approach combined DeepLabV3+[21] with a ResNet50[22] backbone with cross pseudo supervision (CPS),^15^ which we will refer to here on as DeepLabV3 w/CPS. DeepLabV3 w/CPS trains two competing models, CPS_1_ and CPS_2_, on both labeled and unlabeled images (Fig. 2). The training protocol used for the two models was the same as described in the original article.^15^ Both models are the same but were randomly initialized with different weights. The intuition behind using two competing models is that the different initializations allow the models to be different and explore a richer feature space on the labeled images. Next when an unlabeled input image is encountered during training, the output of one model can be used as a pseudo ground truth mask by the other model. The two models cross-supervise each other on unlabeled data through pseudo masks while they both independently learn from labeled data. The cross-supervision enforces regularization on the features learnt by the two competing models. The cross-pseudo supervision loss (referred to from here on as CPS loss) is the sum of a labeled and an unlabeled loss, each of which is composed of two different cross-entropy losses. The labeled loss is the sum of the cross-entropy loss of CPS1 and CPS2, respectively, on a labeled input image and ground truth pair. The unlabeled loss is the sum of the cross-entropy loss from CPS1’s pseudo ground truth mask on an unlabeled image being compared against CPS2’s prediction and the cross-entropy loss from CPS2’s pseudo ground truth mask on an unlabeled image being compared against CPS1’s prediction.

The formula for CPS loss is given below:

(1) $$\begin{array}{ccc} & & \;L_{labeled}\left(X_{i},Y_{i}\right)=L_{CE}\left(CPS_{1}\left(X_{i}\right),Y_{i}\right)\\ & & \;+\;L_{CE}\left(CPS_{2}\left(X_{i}\right),Y_{i}\right)\; \end{array}$$

(2) $$\begin{array}{ccc} & & \;L_{Unlabeled}\left(X_{i}\right)=L_{CE}\left(CPS_{1}\left(X_{i}\right),CPS_{2}\left(X_{i}\right)\right)\\ & & \;+\;L_{CE}\left(CPS_{2}\left(X_{i}\right),CPS_{1}\left(X_{i}\right)\right) \end{array}$$

(3) $$\begin{array}{c} L_{CPS}=L_{Labeled}+L_{Unlabeled} \end{array}$$

where *L_labeled_*(*X_i_*,*Y_i_*) is the sum of the cross-entropy loss of CPS_1_ and CPS_2_ on the *i*th labeled pair of input image and ground truth. *L_CE_*(*CPS*_1_(*X_i_*), *Y_i_*) represents the cross-entropy loss of CPS_1_’s prediction on an input image and *L_CE_*(*CPS*_2_(*X_i_*), *Y_i_*) represents the cross-entropy loss of CPS_2_’s prediction on an input image. *L_Unlabeled_*(*X_i_*) is the sum of the cross-entropy losses of both component models’ respective predictions. *L_CE_*(*CPS*_1_(*X_i_*), *CPS*_2_(*X_i_*)) represents the cross-entropy loss of CPS_1_’s prediction using CPS_2_’s prediction as a pseudo ground truth and *L_CE_*(*CPS*_2_(*X_i_*), *CPS*_1_(*X_i_*)) represents the cross-entropy loss of CPS_2_’s prediction using CPS_1_’s prediction as a pseudo ground truth. The total cross-pseudo supervision loss is given by *L_CPS_* and is the sum of *L_Unlabeled_* and *L_Labeled_*. Once trained, CPS_1_ is used for prediction. Ablation was done on varying amounts of unlabeled data (100%, 75%, 50%, 25%, and no unlabeled data). The second semisupervised model was the Standard UNet with Cross Pseudo Supervision. The training protocol was the same as described above but used a Standard UNet model rather than DeepLabV3 with Resnet. This model is referred to here on as Standard UNet w/CPS. Standard UNet w/CPS was trained with 100% of the unlabeled data. A third semisupervised model combined CPS with the state-of-the-art model, DConnNet, which we will refer to here on as DConnNet w/CPS. The training protocol was the same as described above but used a DConnNet model rather than DeepLabV3 with ResNet and the DConn loss function rather than cross entropy loss. Further information on the training protocol can be found in Supplemental Materials.

A fourth semisupervised model was trained using DeepLabV3 with a ResNet50 backbone but using the Mean Teacher semisupervised framework,^14^ which we denote DeepLabV3 w/MT to serve as a semisupervised baseline, shown in Supplementary Figure S3. DeepLabV3 w/MT trains a student model MT_1_ on both unlabeled and labeled images, and a teacher model MT_2_, on only labeled images (Supplementary Fig. S3). The training protocol used for the two models was the same as described in the original article.^14^ When a labeled image is passed through, the output from the student model is compared against the output from the teacher model and the ground truth and used to backpropagate the student model. The teacher model is updated as an exponential moving average of the student's weights. When an unlabeled image is passed through, this protocol is the same except that the student model does not compare its output against a ground truth. DeepLabV3 w/MT was trained using 100% of the unlabeled data.

### Evaluation

All models were modified and tuned to perform segmentation of the retinal features. We note that the deep learning models learned to segment 13 features from the input images. For the diseased images only, a fourteenth feature, cysts, was added during post-processing (Supplemental Materials). After this post-processing step the model outputs were then compared in terms of the Intersection over Union (IOU) metric on the test dataset against each other and were also compared to the automatically generated segmentations from the baseline Heidelberg automatic model. To evaluate each of the models, the IOU were calculated for each model's post-processed final prediction for 74 diseased test images and 140 non-pathology test images. Demographics of test data is given in Supplemental Table S1. We compared the IOUs of the six supervised models and the eight semisupervised baseline models, as well as Heidelberg Auto to our full model, DeepLabV3 w/CPS–100%, by testing if our model outperformed every other model in each of the 14 segmented or postprocessed classes using a one-sided paired signed-rank test. The Heidelberg Auto baseline is only preprocessed to extract the layer boundaries and not post-processed to reflect how the automatic segmenter on Heidelberg devices handles diseased retinas.

## Results

**Figure 3.**
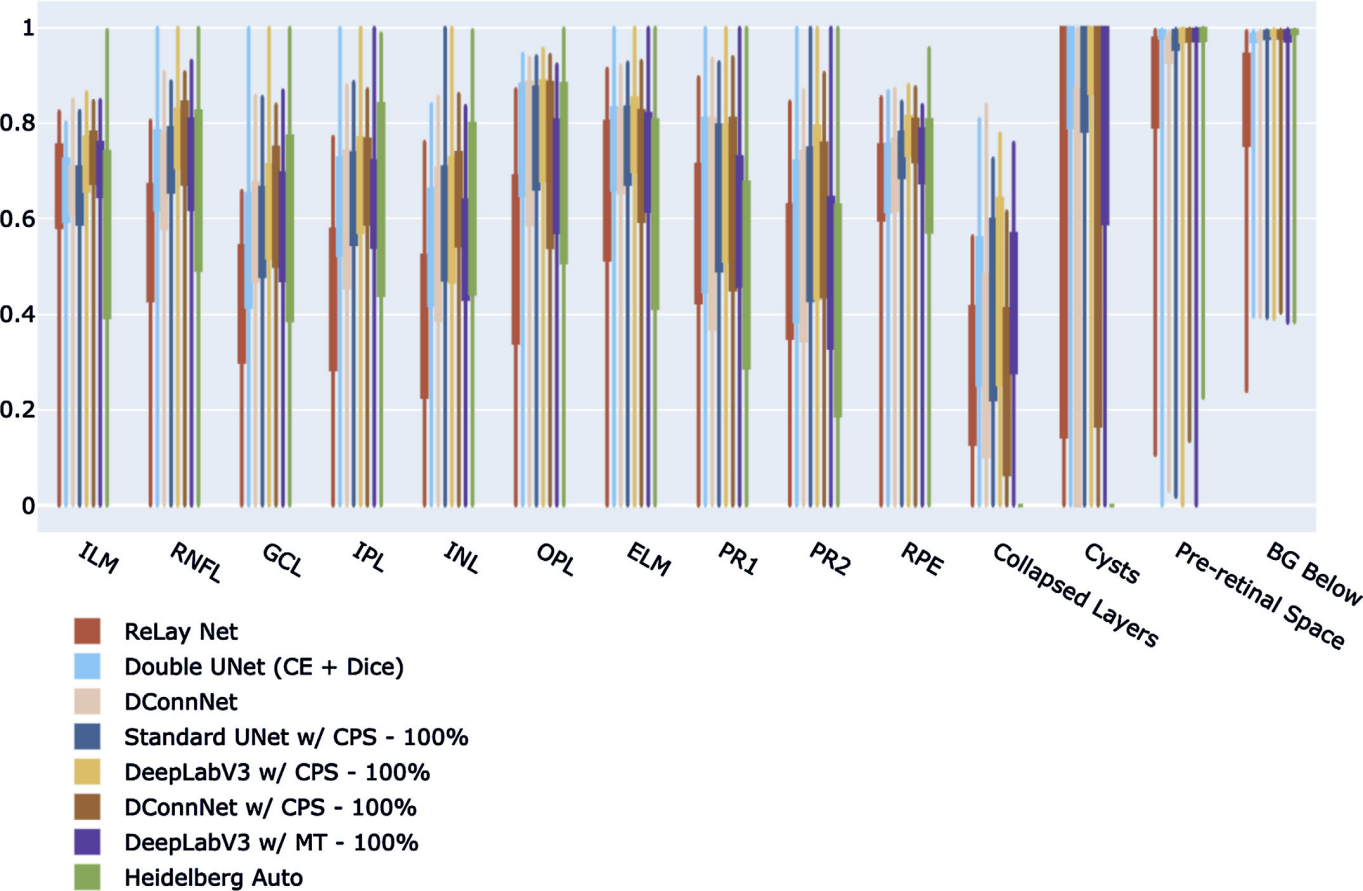
IOU comparison of the supervised models, unsupervised models, and Heidelberg Auto on the diseased test set. For each semisupervised model (DeepLabV3 w/CPS, DeepLabV3 w/MT, Standard UNet w/CPS), the highest performing version is shown, where 100% of the unlabeled data was used in training.

In Figure 3, we show the range of IOUs for each class for two popular supervised models (RelayNet, DConnNet), the best supervised model, Double UNet (CE + Dice), versus the semisupervised models using all the unlabeled data (UNet w/CPS–100%, DeepLabV3 w/CPS–100%, DConnNet w/CPS–100%, DeepLabV3 w/MT–100%, ) versus the baseline model Heidelberg Auto on the diseased test set. The semisupervised models generally have higher IOUs than the supervised models and the baseline Heidelberg Auto. For completeness, Table 1 shows the mean IOUs and confidence intervals on all 14 features for the 14 trained models and the baseline Heidelberg Auto model on the diseased test set. Supplementary Table S3 shows similar results on the non-pathology test set. Our full model, DeepLabV3 w/CPS–100%, has a higher median IOU for each class versus the other models. In addition, we systematically compare our best model, DeepLabV3 w/CPS–100%, to the other 14 models in terms of IOU for each class on the 74 test images. The mean difference in IOUs by class for each of the 74 test images of each of the other 14 models to our model DeepLabV3 w/CPS–100%, is shown in Table 2. IOUs that are significantly positive by a one-sided paired signed rank test, that is where DeepLabV3 w/CPS–100% significantly outperformed, are marked with an asterisk. The corresponding *P* values table is given in Supplemental Table S2. We note that our model DeepLabV3 w/CPS–100%, significantly outperformed previously notable supervised models such as RelayNet, UNet (CE + Dice), and DConnNet on every class, as well as a previous semisupervised model DeepLabV3 w/MT– 100% on all but the ILM and preretinal space. Supplementary Figures S6 and S7 show a comparison of the supervised models only on the diseased and non-pathology test set. Supplementary Figure S9 shows sample segmentations of each of these respective models on the diseased test set.

**Table 1.** The Average IOU and Standard Error Per Layer for Each Model on the Diseased Test Set Are Shown


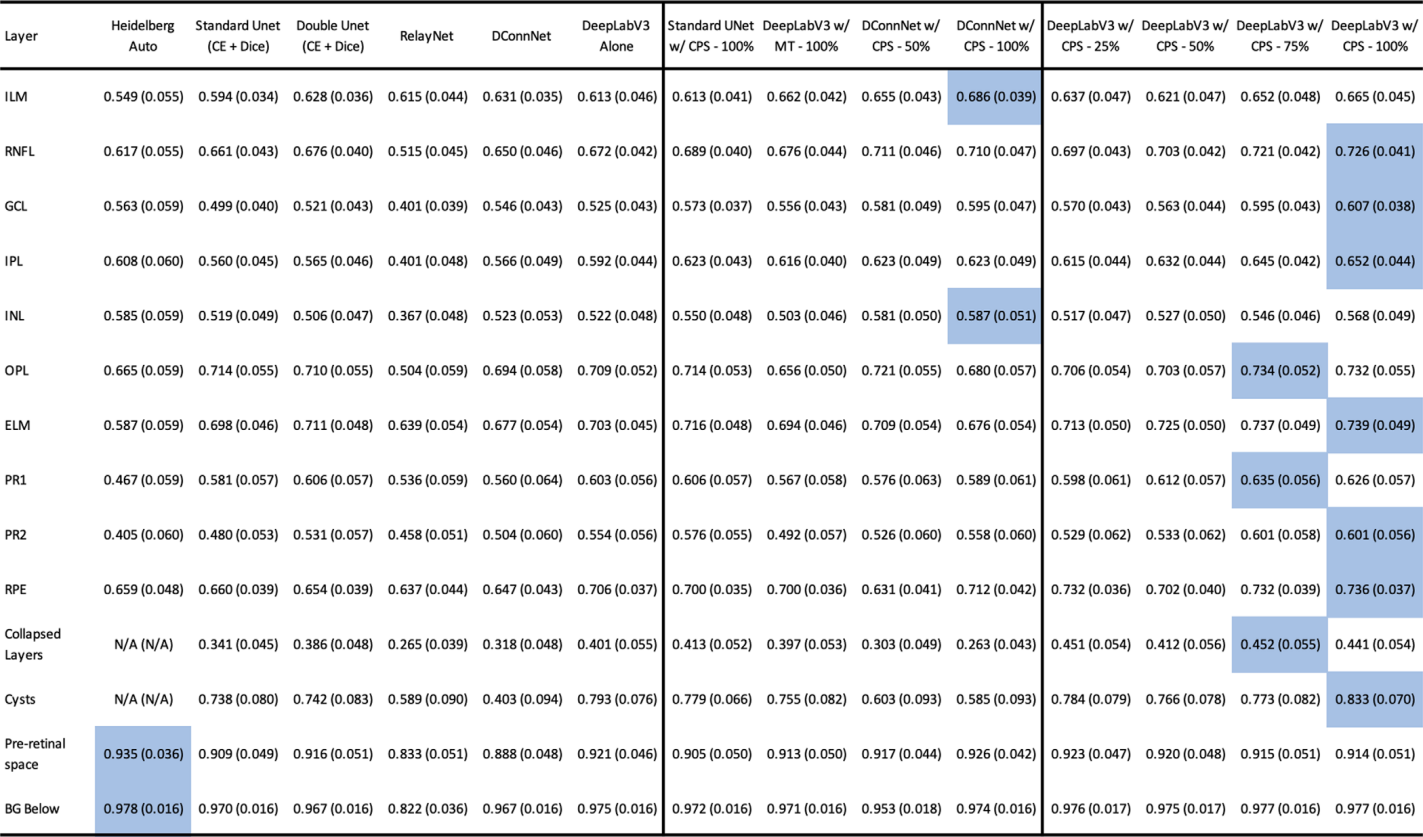

ELM, external limiting membrane; GCL, ganglion cell layer; INL, inner nuclear layer; IPL, inner plexiform layer; OPL, outer plexiform layer; PR1, photoreceptor 1; PR2, photoreceptor 2; RNFL, retinal nerve fiber layer; preretinal space, and background below RPE.

The Highest Mean IOU of Each Row is Highlighted in Blue.

* The highest mean IOU of the row.

**Table 2.** Mean of Difference in IOUs on the Diseased Test Set by Layer for Each Test Image Versus DeepLabV3 w/CPS - 100%


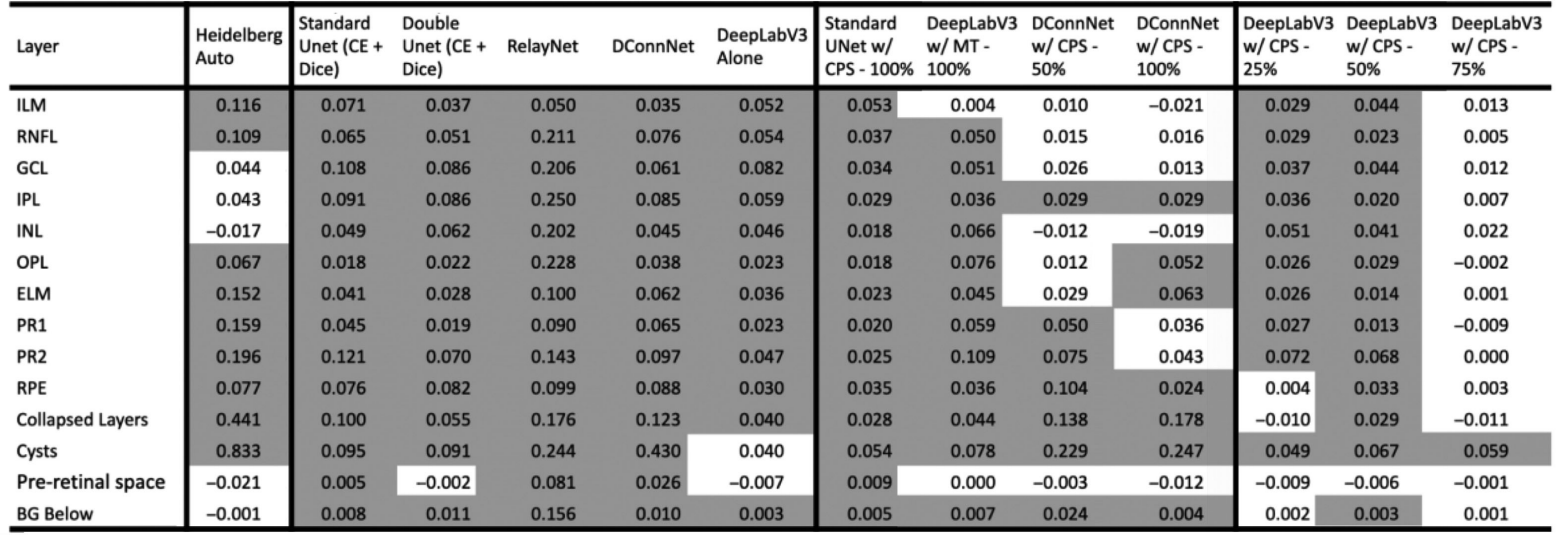

BG, background; ELM, external limiting membrane; GCL, ganglion cell layer; INL, inner nuclear layer; IPL, inner plexiform layer; OPL, outer plexiform layer; PR1, photoreceptor 1; PR2, photoreceptor 2; RNFL, retinal nerve fiber layer.

The *P* values are provided in Supplementary Table S2.

Significantly Different IOUs are Highlighted In Gray.

* Significantly different IOU.

Next, we analyze the benefit of using unlabeled data, by comparing models that use progressively more unlabeled data using the same segmenter. For example, DeepLabV3 w/CPS–25%, DeepLabV3 w/CPS–50%, DeepLabV3 w/CPS–75%, and DeepLabV3 w/CPS–100% all use CPS, but different amounts of unlabeled data, and can be compared to DeepLabV3 alone, which is a supervised model that uses no unlabeled data (Supplementary Fig. S4, Supplemental Fig. S5 and Supplementary Table S1). From Table 2, we see that the mean difference in IOUs by class decreases for the majority of the classes as more unlabeled data is used. This trend is corroborated by DConnNet w/CPS using different amounts of unlabeled data on the diseased test set and by statistical tests, as DeepLabV3 w/CPS–100% significantly outperforms DeepLabV3 alone on all but the preretinal space class. Then DeepLabV3 w/CPS–100% outperforms DeepLabV3 w/CPS–25% on all but the RPE, collapsed layers, preretinal space, and BG below. Finally, DeepLabV3 w/CPS–100% only outperforms DeepLabV3 w/CPS–75% on the Cysts class. This confirms that more unlabeled data improves segmentation performance, with some decreasing returns as more unlabeled data is incorporated (Supplementary Fig. S8).

For nonpathological images, the semisupervised methods generally outperformed the supervised methods, Supplementary Table S3. In addition, DeepLab V3 w/CPS–100% was the best performing semisupervised model, achieving the top or joint top IOU on seven out of 14 retinal features.

**Figure 4.**
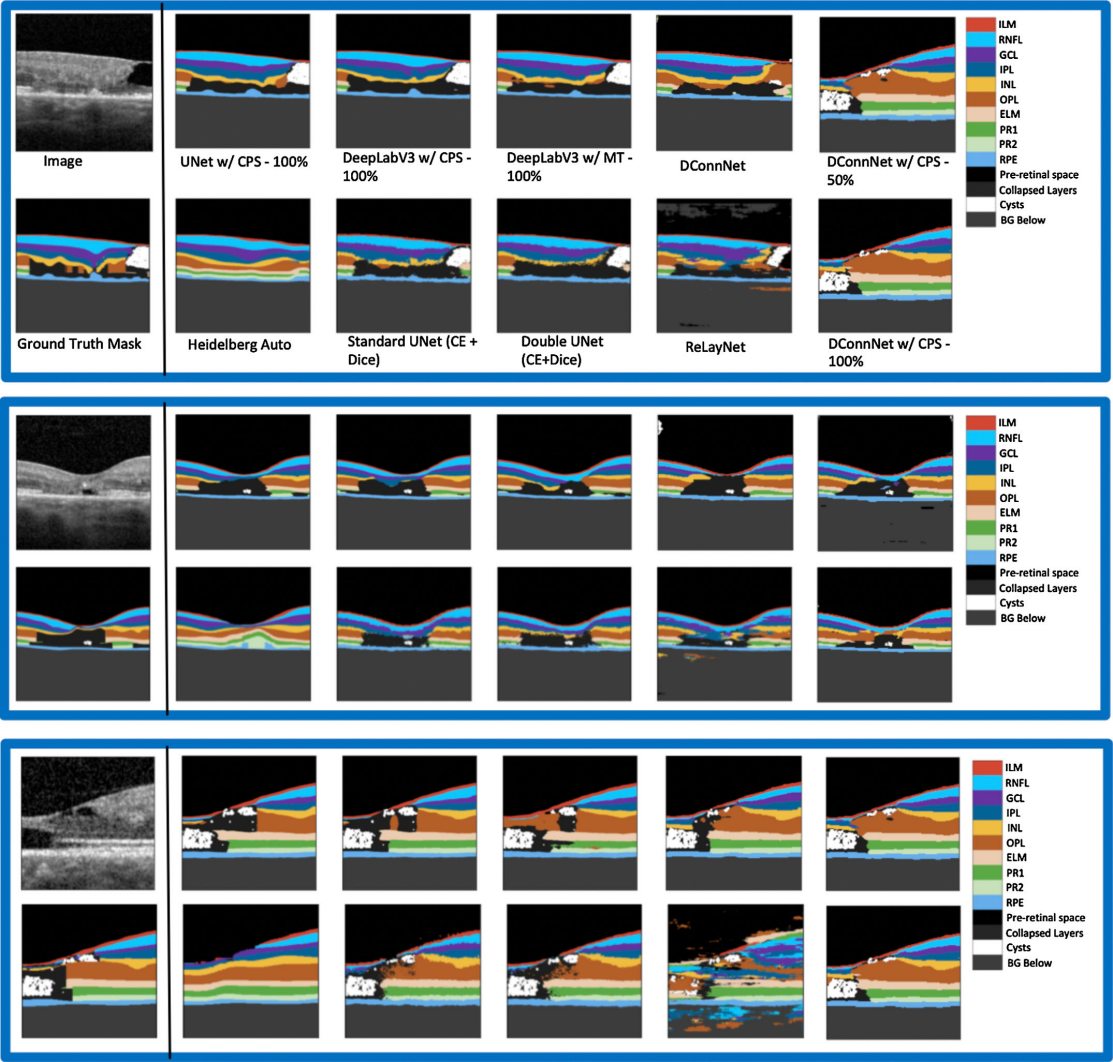
Sample post-processed predictions of different models on the diseased test set. For each set of segmentations shown above from left to right, starting from the top row is the input image, UNet w/CPS prediction, the DeepLabV3 w/CPS prediction, and the DeepLabV3 w/MT prediction. From left to right on the bottom row is the Ground Truth Mask, the Heidelberg Auto prediction, the Standard UNet (CE + Dice loss) prediction, the Double UNet (CE + Dice loss) prediction, and the ReLayNet prediction.

Sample segmentations of the models on the test images are shown in Figure 4. DeepLabV3 w/CPS–100%'s predictions are much smoother than the predictions of the models. More generally, the semisupervised models tend to have smoother predictions than the supervised models. The manufacturer model, Heidelberg Auto, fails to detect anomalies and interpolated smooth retinal layers for the pathological eyes in the test dataset.

**Figure 5.**
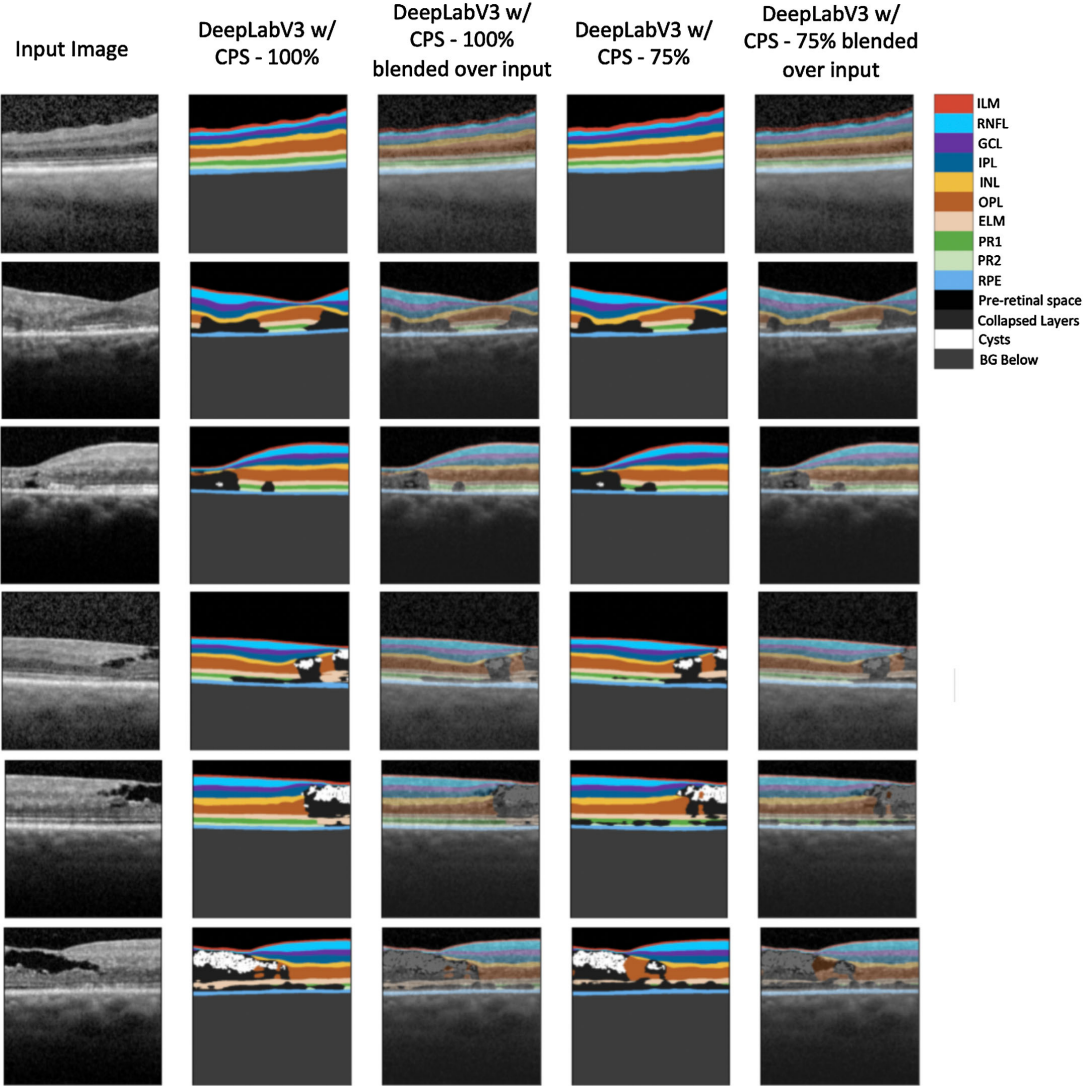
DeepLabV3 w/CPS–100% and DeepLabV3 w/CPS–75% sample segmentations on DICOM files. Shown above from left to right is the input image, DeepLabV3 w/CPS–100% prediction, DeepLabV3 w/CPS–100% prediction blended over the input, DeepLabV3 w/CPS–75% prediction, and DeepLabV3 w/CPS–75% prediction blended over the input.

Finally, Figure 5 shows the predictions of DeepLabV3 w/CPS–100% on DICOM images are robust and generalizable to DICOM array data, while it was trained on screenshots and not DICOM array data. No ground truth is available for these DICOM images because Heidelberg Auto could not be run.

## Discussion

We used semisupervised learning to develop a segmentation algorithm, DeepLabV3 w/CPS–100%. This semisupervised model achieved the best IOU on almost all classes. It statistically significantly outperformed supervised models, such as the Double UNet, and the DeepLabV3 alone and DConnNet. It also statistically significantly outperformed the baseline Heidelberg Auto on seven out of 10 retinal layers. In addition, DeepLabV3 w/CPS–100% outperformed seven other semisupervised models.

Our ablation studies of semisupervised learning frameworks, such as CPS and MT, yields insights in the application of semisupervised learning to the segmentation of retinal layers. DeepLabV3 w/CPS–100% and the other semisupervised models outperformed the supervised methods, Standard UNet, Double UNet, ReLayNet, DeepLabV3, and DConnNet alone on all 10 layers and all four features. The semisupervised models successfully leveraged the 13308 unlabeled input images to improve segmentation. A previous study to improve supervised deep learning methods in uncommon diseases, such as MacTel, sought to increase the set of labeled data by including synthetic OCT images from generative deep learning models.^23^ However, the authors of that article focused on classification and noted that many of the generated OCT images contained artifacts, which would make them unsuitable for a segmentation task. Our result shows that semisupervised learning can significantly improve segmentation, especially where labeled data is sparse, which is often the case in rare diseases.

Next, we analyzed the benefit of different amounts of unlabeled data by comparing DeepLabV3 w/CPS–100% to DeepLabV3 w/CPS–25%, DeepLabV3 w/CPS–50%, and DeepLabV3 w/CPS–75%. We found that DeepLabV3 w/CPS–100% significantly outperformed DeepLabV3 w/CPS–25% and DeepLabV3 w/CPS–50% on most layers but was not significantly better than DeepLabV3 w/CPS–75%. This shows there are diminishing returns to using unlabeled data. The quality of DeepLabV3 w/CPS–100%’s segmentation can be assessed qualitatively. DeepLabV3 w/CPS–100% were less susceptible than DeepLabV3 w/CPS trained on lower amounts of unlabeled data to have interrupted gaps in its layers.

Finally, we analyzed the choice of segmentation networks and semisupervised frameworks, by comparing our DeepLabV3 w/CPS–100% to DeepLabV3 w/MT–100%, Standard UNet w/CPS–100% and DConnNet w/CPS–100%. Our DeepLabV3 w/CPS–100% statistically significantly outperformed DeepLabV3 w/MT–100% on all but the ILM and preretinal space classes. Intuitively this makes sense because the CPS framework has two distinct models cross-supervise each other, whereas MT essentially explores with the student model that slowly updates the teacher, which restricts the breadth of data representations in comparison to CPS. Next, our DeepLabV3 w/CPS–100% statistically significantly outperformed Standard UNet w/CPS–100% on all classes, and DConnNet w/CPS–100% on 7/14 classes and was not significantly different on the remaining classes, suggesting that the choice of segmenter is important in maximizing the performance of the semisupervised framework.

We demonstrated the superior performance of DeepLabV3 w/CPS–100% on segmenting the retinal features versus popular supervised models, but this approach has some limitations. First, given the difficulty in acquiring manually segmented ground truth masks, DeepLabV3 w/CPS–100% and the supervised models were evaluated on only diseased 74 test and 140 non-pathological images. The small number of test images may not capture all the different anatomical variations that can occur in patients’ eyes. Further validation is needed on a larger dataset with more labeled test examples before this model could be applied in clinical care. Second, DeepLabV3 w/CPS–100% was trained only on scans taken using the Heidelberg Spectralis device and may not generalize to scans from other devices such as the Zeiss Cirrus (Zeiss, Oberkochen, Germany) or Topcon (Topcon Optical Company, Tokyo, Japan) OCT devices. Finally, our model does not segment the Bruch's membrane and could be explored in future work.

In conclusion, DeepLabV3 w/CPS–100% is a segmentation algorithm that leverages unlabeled data and improves segmentation performance, especially of disrupted layers, in MacTel. The accurate segmentation of retinal layers and features can help define characteristics of different disease stages of MacTel in OCT. The semisupervised approach taken can be applied to improve feature identification and location to other disease, where there is a small number of labeled images and a large number of unlabeled images.

## Supplementary Material

Supplement 1

Supplement 2

Supplement 3

Supplement 4

Supplement 5

Supplement 6

Supplement 7

Supplement 8

Supplement 9

Supplement 10

Supplement 11

Supplement 12

Supplement 13
