# Supplementary material for: Developing a 10-Layer Retinal Segmentation for MacTel Using Semi-Supervised Learning
Source: Transl Vis Sci Technol. 2024 Nov 5;13(11):2. doi: 10.1167/tvst.13.11.2 (PMC11542501; doi:10.1167/tvst.13.11.2)
Supplement: Supplement 2 [file tvst-13-11-2_s002.pdf]

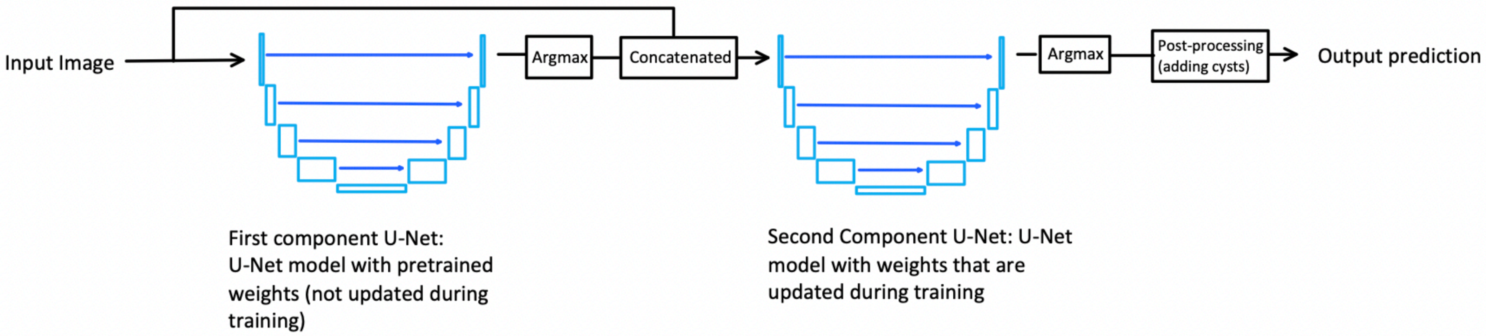

Fig. S2. Schematic for Double U-Net. Input image is passed through the first component U-Net, and its predicted output is concatenated with the initial input image and then passed through the second component U-Net to produce a prediction. This prediction is post-processed for cysts to generate the final prediction.
