# Supplementary material for: Developing a 10-Layer Retinal Segmentation for MacTel Using Semi-Supervised Learning
Source: Transl Vis Sci Technol. 2024 Nov 5;13(11):2. doi: 10.1167/tvst.13.11.2 (PMC11542501; doi:10.1167/tvst.13.11.2)
Supplement: Supplement 3 [file tvst-13-11-2_s003.pdf]

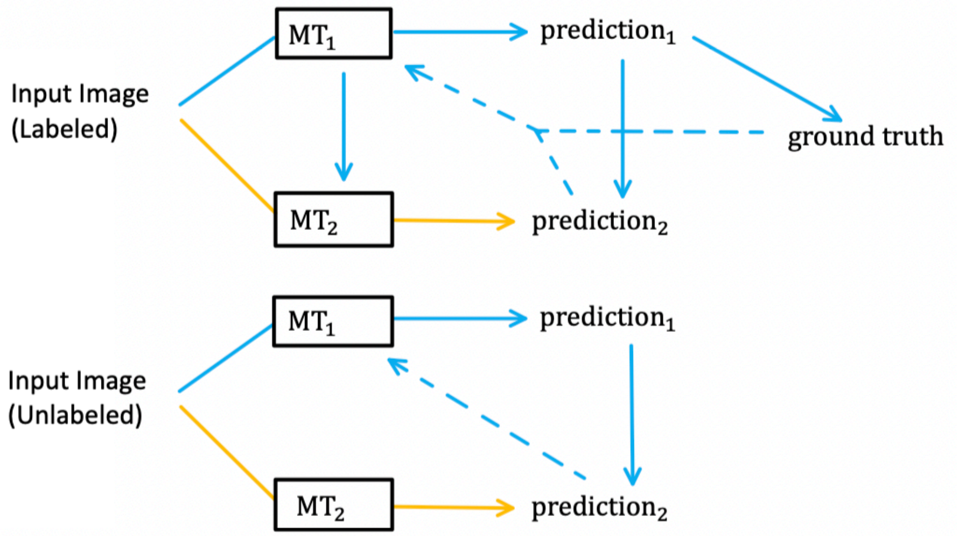

Fig S3. Schematic for DeepLabV3 w/ MT. When a labeled image is passed through, the ground truth and output from the teacher model are used to backpropagate the student model. When an unlabeled image is passed through, the teacher's output is used to backpropagate the student model. In both cases, the teacher's model weights are an exponential moving average of the student's model weights.
