# Supplementary material for: Developing a 10-Layer Retinal Segmentation for MacTel Using Semi-Supervised Learning
Source: Transl Vis Sci Technol. 2024 Nov 5;13(11):2. doi: 10.1167/tvst.13.11.2 (PMC11542501; doi:10.1167/tvst.13.11.2)
Supplement: Supplement 4 [file tvst-13-11-2_s004.pdf]

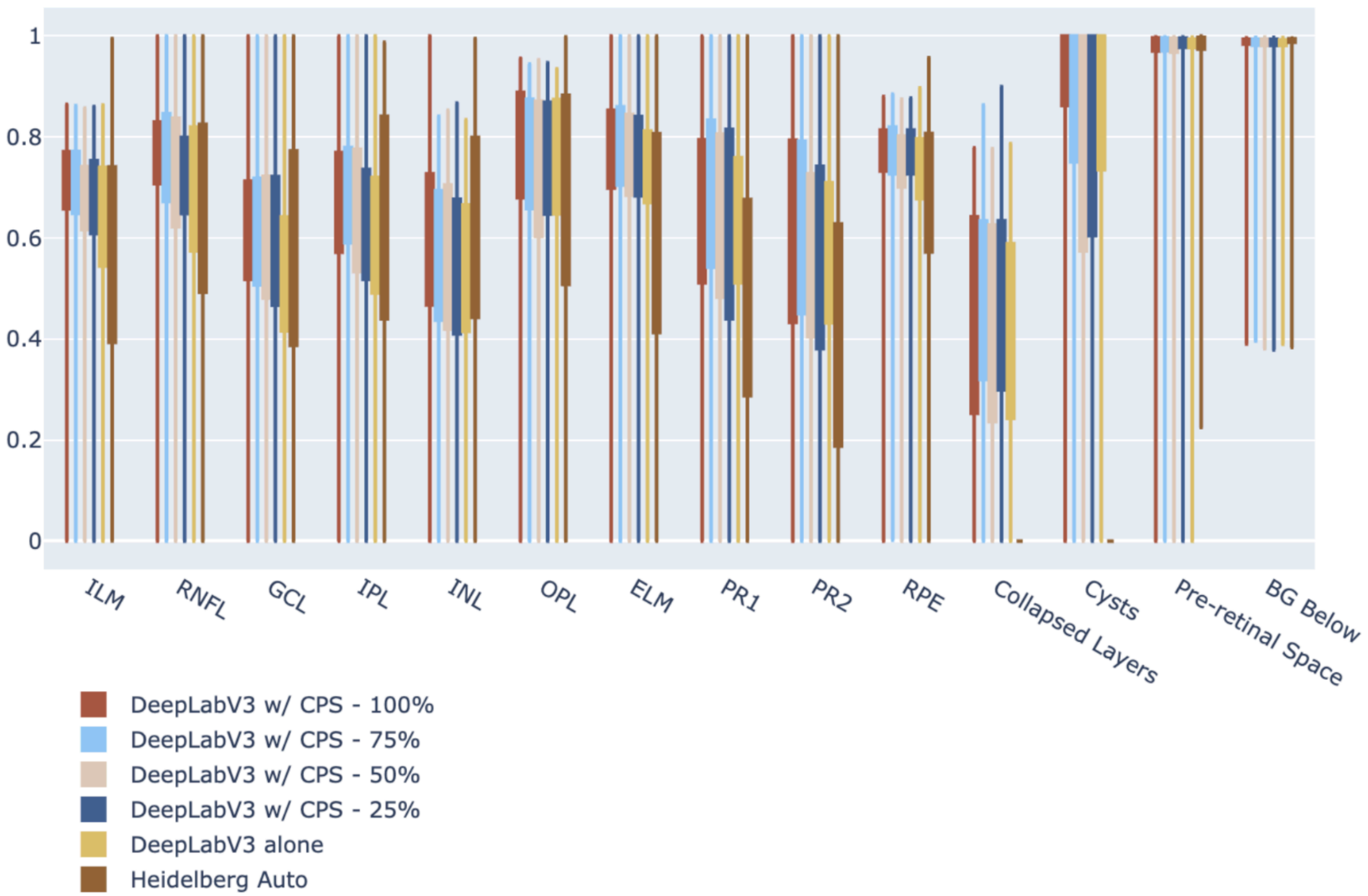

Fig. S4. IOU performance on the performance on the diseased test set by DeepLabV3 w/ CPS - 100%, DeepLabV3 w/ CPS - 75% , DeepLabV3 w/ CPS - 50%, DeepLabV3 w/ CPS - 25%, DeepLabV3 alone, and Heidelberg Auto. The percentages indicate the amounts of unlabeled data utilized by the respective model.
