# Supplementary material for: Developing a 10-Layer Retinal Segmentation for MacTel Using Semi-Supervised Learning
Source: Transl Vis Sci Technol. 2024 Nov 5;13(11):2. doi: 10.1167/tvst.13.11.2 (PMC11542501; doi:10.1167/tvst.13.11.2)
Supplement: Supplement 6 [file tvst-13-11-2_s006.pdf]

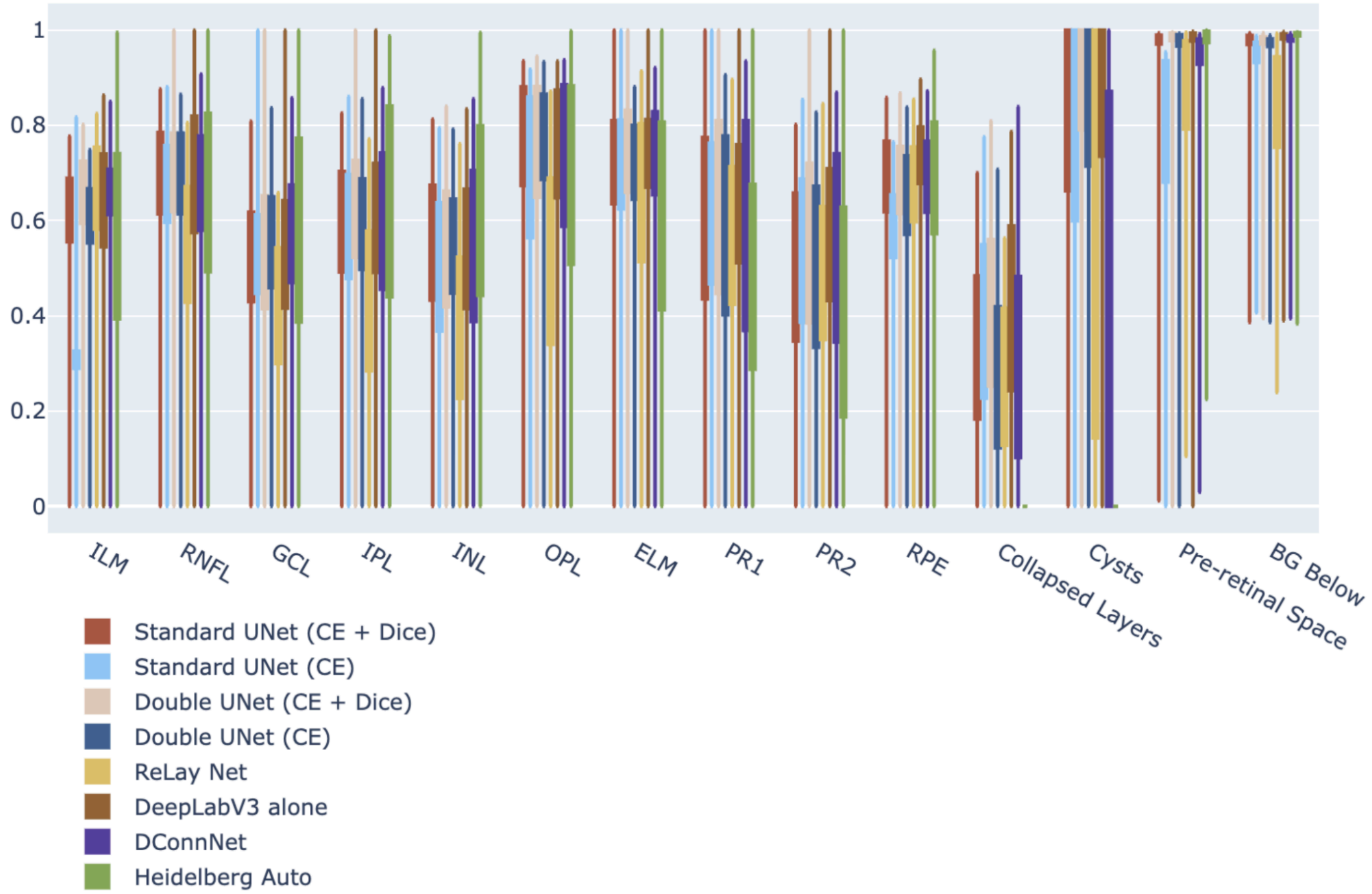

Fig. S6. Comparison of the IOU per layer for different supervised models on the diseased test set by layer
