# Supplementary material for: Developing a 10-Layer Retinal Segmentation for MacTel Using Semi-Supervised Learning
Source: Transl Vis Sci Technol. 2024 Nov 5;13(11):2. doi: 10.1167/tvst.13.11.2 (PMC11542501; doi:10.1167/tvst.13.11.2)
Supplement: Supplement 8 [file tvst-13-11-2_s008.pdf]

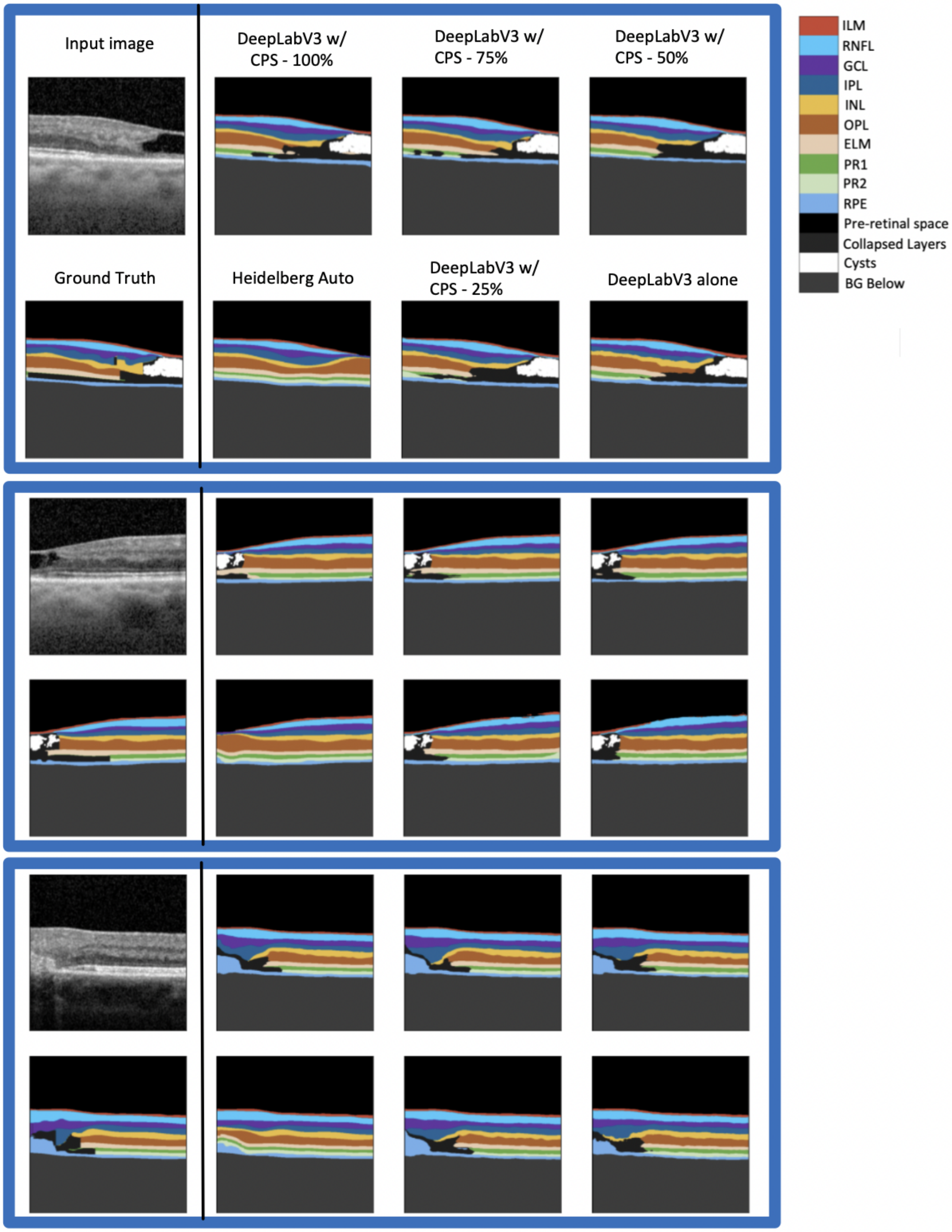

Fig. S8. Sample post-processed predictions of DeepLabV3 w/ CPS trained on different amounts of unlabeled data on the diseased test set. For each set of segmentations shown above from left to right, starting from the top row is the input image, DeepLabV3 w/ CPS - 100% prediction, DeepLabV3 w/ CPS - 75% prediction, and DeepLabV3 w/ CPS - 50% prediction. For the bottom row, from left to right is the ground truth mask, Heidelberg Auto prediction, DeepLabV3 w/ CPS - 25% prediction,and DeepLabV3 alone prediction. The percentages indicate how much of the unlabeled data they utilized in training.
