# Supplementary material for: Developing a 10-Layer Retinal Segmentation for MacTel Using Semi-Supervised Learning
Source: Transl Vis Sci Technol. 2024 Nov 5;13(11):2. doi: 10.1167/tvst.13.11.2 (PMC11542501; doi:10.1167/tvst.13.11.2)
Supplement: Supplement 9 [file tvst-13-11-2_s009.pdf]

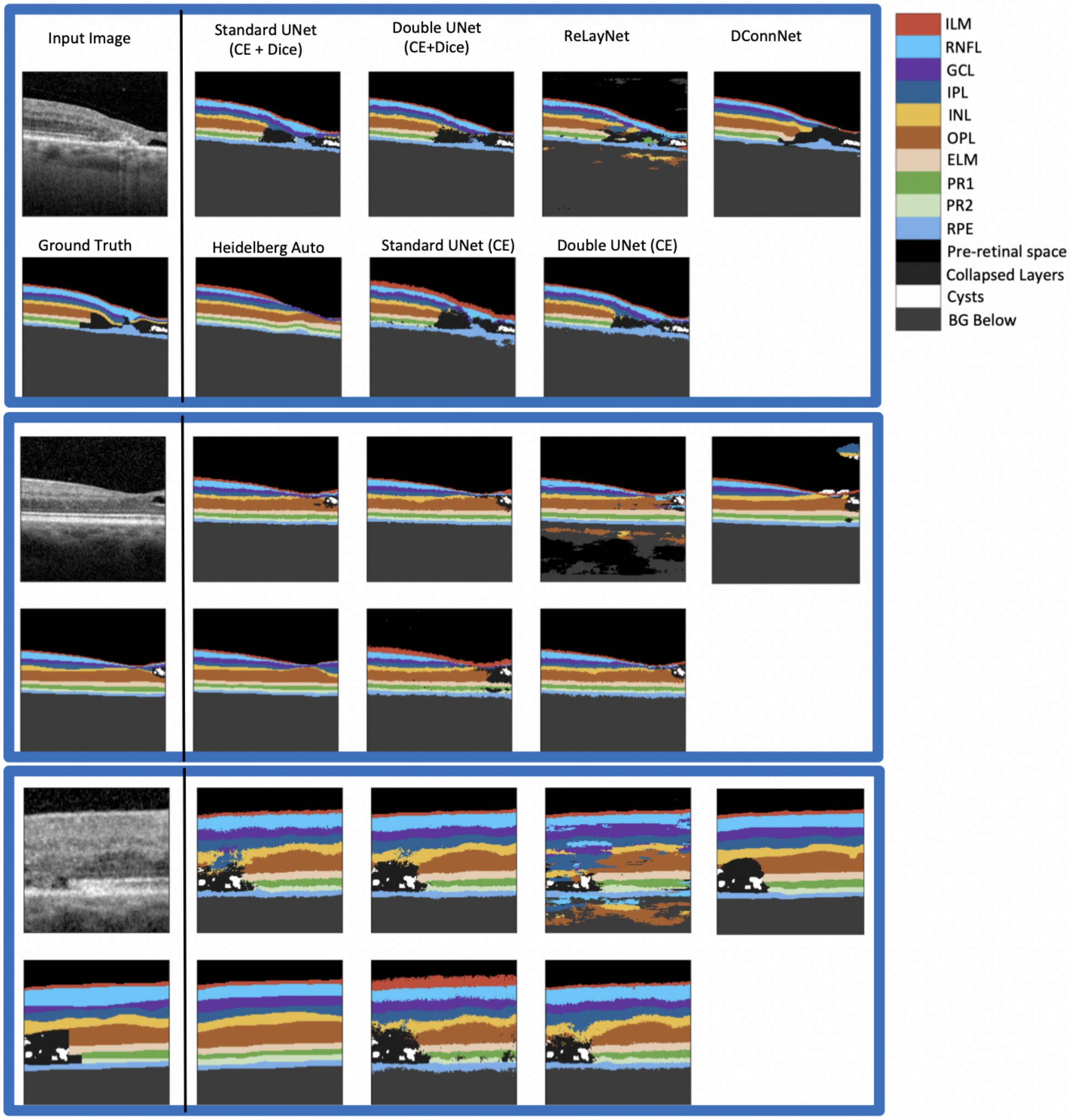

Fig. S9. Sample post-processed segmentations of Supervised models on diseased test set. For each set of segmentations shown above, from left to right, on the top row, is the input image, Standard UNet with Cross Entropy plus Dice loss function (CE + Dice) prediction, Double UNet (CE + Dice) prediction, and ReLayNet prediction. On the bottom row, from left to right, is the ground truth mask, Heidelberg Auto prediction, Standard UNet with Cross Entropy loss (CE) prediction, and Double UNet (CE) prediction.
