# Supplementary material for: Developing a 10-Layer Retinal Segmentation for MacTel Using Semi-Supervised Learning
Source: Transl Vis Sci Technol. 2024 Nov 5;13(11):2. doi: 10.1167/tvst.13.11.2 (PMC11542501; doi:10.1167/tvst.13.11.2)
Supplement: Supplement 10 [file tvst-13-11-2_s010.pdf]

Table S1. Summary of the diseased test set showing the number of images in the diseased test set with cysts and collapsed layers and the non-pathology test set.

Diseased test set	
Total images	74
images with cysts	26
images with collapsed layers	74
**Non-pathology test set**	
Total Images	140
