# Supplementary material for: Developing a 10-Layer Retinal Segmentation for MacTel Using Semi-Supervised Learning
Source: Transl Vis Sci Technol. 2024 Nov 5;13(11):2. doi: 10.1167/tvst.13.11.2 (PMC11542501; doi:10.1167/tvst.13.11.2)
Supplement: Supplement 11 [file tvst-13-11-2_s011.pdf]

Table S2 shows the p-values of a one-sided paired signed rank test by class over the 74 test images for the 12 models versus DeepLabV3 w/ CPS - 100%. Shaded values are values that were significant.

	Heidelberg Auto	Standard UNet (CE + Dice)	Double UNet (CE + Dice)	ReLay Net	DConnNet	DeepLabV3 alone	Standard UNet w/ CPS - 100%	DeepLabV3 w/ MT - 100%	DConnNet w/ CPS - 50%	DConnNet w/ CPS - 100%	DeepLabV3 w/ CPS - 25%	DeepLabV3 w/ CPS - 50%	DeepLabV3 w/ CPS - 75%
ILM	1.35E-04	5.88E-08	1.61E-05	1.16E-04	5.30E-05	1.08E-04	1.53E-11	5.15E-02	6.27E-02	5.03E-01	1.09E-02	1.57E-06	2.54E-01
RNFL	9.39E-06	2.15E-06	3.57E-06	7.25E-13	1.12E-05	9.47E-07	3.87E-12	1.10E-04	1.98E-01	2.42E-01	1.23E-03	7.38E-03	5.44E-01
GCL	7.42E-02	5.65E-09	8.03E-08	8.88E-13	1.28E-04	4.16E-08	1.55E-11	1.81E-04	9.50E-02	4.92E-01	1.43E-03	2.78E-04	2.36E-01
IPL	1.44E-01	1.71E-09	1.94E-08	2.85E-13	1.41E-07	1.82E-07	7.62E-12	1.03E-04	2.45E-02	2.15E-02	2.49E-04	8.22E-03	1.95E-01
INL	9.33E-01	4.23E-04	2.54E-05	1.91E-12	4.03E-03	2.48E-03	5.03E-10	3.14E-04	9.88E-01	9.88E-01	8.62E-04	7.83E-04	5.77E-02
OPL	3.65E-04	1.37E-02	3.21E-03	4.10E-13	1.07E-03	7.43E-04	1.56E-10	8.82E-08	2.02E-01	1.67E-04	1.79E-03	4.58E-04	4.04E-01
ELM	1.45E-08	2.34E-08	4.94E-05	1.01E-09	2.50E-06	5.64E-06	1.50E-10	1.67E-06	7.04E-02	9.61E-07	7.44E-04	1.74E-02	3.91E-01
PR1	4.15E-08	3.49E-06	1.10E-03	2.03E-09	4.42E-04	2.11E-03	7.65E-09	2.57E-06	2.76E-03	1.57E-01	2.06E-02	9.77E-03	6.44E-01
PR2	1.09E-07	1.27E-06	3.09E-04	3.07E-09	2.49E-04	1.23E-03	1.03E-09	1.06E-07	4.84E-03	7.93E-01	2.96E-03	1.37E-04	9.31E-01
RPE	2.41E-04	1.03E-09	2.27E-10	8.39E-11	9.35E-11	4.86E-04	4.31E-12	1.27E-05	9.83E-13	2.21E-02	2.64E-01	3.52E-05	5.06E-01
Collapsed Layers	5.73E-13	1.01E-07	3.89E-04	4.81E-12	3.40E-09	1.47E-02	2.39E-12	1.74E-04	4.39E-09	1.64E-10	5.64E-01	1.33E-02	7.69E-01
Cysts	1.22E-13	6.41E-06	3.54E-03	4.48E-08	8.38E-09	1.16E-01	5.25E-08	8.07E-04	4.24E-08	7.07E-07	4.58E-02	1.43E-02	3.88E-02
Pre-retinal space	9.53E-01	1.12E-08	3.36E-05	1.35E-08	1.06E-07	3.44E-02	4.15E-08	5.13E-02	9.98E-03	8.51E-01	4.89E-01	4.42E-03	5.78E-01
BG Below	9.77E-01	1.28E-11	8.88E-12	7.57E-14	1.60E-09	4.20E-05	3.12E-12	2.92E-05	4.90E-12	1.27E-04	2.17E-01	8.02E-03	6.27E-01
