# Supplementary material for: Developing a 10-Layer Retinal Segmentation for MacTel Using Semi-Supervised Learning
Source: Transl Vis Sci Technol. 2024 Nov 5;13(11):2. doi: 10.1167/tvst.13.11.2 (PMC11542501; doi:10.1167/tvst.13.11.2)
Supplement: Supplement 12 [file tvst-13-11-2_s012.pdf]

Table S3: The average IOU and standard error per layer for each model on the non-pathology test set are shown. The highest mean IOU of each row is highlighted in blue

| Layer | Standard Unet (CE + Dice) | Double Unet (CE + Dice) | RelayNet | DConnNet | DeepLabV3 Alone | Standard UNet w/ CPS - 100% | DeepLabV3 w/ MT - 100% | DConnNet w/ CPS - 50% | DConnNet w/ CPS - 100% | DeepLabV3 w/ CPS - 25% | DeepLabV3 w/ CPS - 50% | DeepLabV3 w/ CPS - 75% | DeepLabV3 w/ CPS - 100% |
|---|---|---|---|---|---|---|---|---|---|---|---|---|---|
| ILM | 0.137 (0.034) | 0.136 (0.034) | 0.135 (0.034) | 0.163 (0.036) | 0.301 (0.039) | 0.355 (0.018) | 0.402 (0.041) | 0.152 (0.036) | 0.130 (0.035) | 0.361 (0.044) | 0.329 (0.043) | 0.376 (0.047) | 0.415 (0.049) |
| RNFL | 0.492 (0.035) | 0.521 (0.036) | 0.479 (0.030) | 0.511 (0.035) | 0.616 (0.037) | 0.522 (0.026) | 0.616 (0.039) | 0.492 (0.035) | 0.475 (0.035) | 0.626 (0.037) | 0.638 (0.038) | 0.629 (0.044) | 0.643 (0.044) |
| GCL | 0.513 (0.035) | 0.548 (0.036) | 0.557 (0.033) | 0.564 (0.035) | 0.613 (0.037) | 0.506 (0.031) | 0.597 (0.039) | 0.507 (0.036) | 0.490 (0.036) | 0.615 (0.037) | 0.616 (0.037) | 0.614 (0.041) | 0.624 (0.041) |
| IPL | 0.580 (0.032) | 0.590 (0.032) | 0.544 (0.033) | 0.580 (0.033) | 0.621 (0.031) | 0.502 (0.026) | 0.605 (0.033) | 0.547 (0.033) | 0.533 (0.033) | 0.607 (0.034) | 0.604 (0.032) | 0.615 (0.035) | 0.621 (0.035) |
| INL | 0.531 (0.030) | 0.541 (0.031) | 0.488 (0.033) | 0.535 (0.032) | 0.582 (0.031) | 0.553 (0.027) | 0.567 (0.031) | 0.508 (0.033) | 0.512 (0.033) | 0.584 (0.033) | 0.550 (0.035) | 0.572 (0.035) | 0.588 (0.036) |
| OPL | 0.804 (0.025) | 0.811 (0.026) | 0.692 (0.032) | 0.797 (0.025) | 0.818 (0.025) | 0.801 (0.025) | 0.788 (0.024) | 0.796 (0.025) | 0.793 (0.025) | 0.820 (0.027) | 0.813 (0.025) | 0.816 (0.027) | 0.815 (0.028) |
| ELM | 0.655 (0.034) | 0.655 (0.034) | 0.666 (0.035) | 0.674 (0.036) | 0.642 (0.035) | 0.635 (0.029) | 0.604 (0.035) | 0.661 (0.035) | 0.665 (0.035) | 0.663 (0.035) | 0.642 (0.036) | 0.650 (0.035) | 0.647 (0.035) |
| PR1 | 0.667 (0.032) | 0.665 (0.033) | 0.654 (0.032) | 0.676 (0.035) | 0.665 (0.033) | 0.651 (0.030) | 0.628 (0.035) | 0.669 (0.034) | 0.663 (0.034) | 0.671 (0.033) | 0.650 (0.035) | 0.665 (0.033) | 0.649 (0.032) |
| PR2 | 0.550 (0.034) | 0.565 (0.035) | 0.489 (0.034) | 0.565 (0.036) | 0.531 (0.035) | 0.554 (0.028) | 0.502 (0.037) | 0.560 (0.035) | 0.577 (0.037) | 0.522 (0.036) | 0.502 (0.036) | 0.533 (0.037) | 0.508 (0.037) |
| RPE | 0.595 (0.032) | 0.622 (0.032) | 0.606 (0.035) | 0.585 (0.033) | 0.629 (0.030) | 0.537 (0.026) | 0.598 (0.033) | 0.638 (0.033) | 0.624 (0.035) | 0.587 (0.034) | 0.580 (0.032) | 0.625 (0.031) | 0.617 (0.032) |
| Pre-retinal space | 0.783 (0.031) | 0.786 (0.031) | 0.607 (0.038) | 0.802 (0.027) | 0.843 (0.026) | 0.792 (0.033) | 0.851 (0.029) | 0.796 (0.029) | 0.787 (0.029) | 0.853 (0.028) | 0.830 (0.030) | 0.847 (0.028) | 0.863 (0.025) |
| BG Below | 0.943 (0.012) | 0.948 (0.011) | 0.761 (0.022) | 0.939 (0.012) | 0.951 (0.011) | 0.915 (0.011) | 0.943 (0.012) | 0.952 (0.012) | 0.946 (0.012) | 0.946 (0.012) | 0.949 (0.012) | 0.953 (0.010) | 0.953 (0.011) |
