# Supplementary material for: Developing a 10-Layer Retinal Segmentation for MacTel Using Semi-Supervised Learning
Source: Transl Vis Sci Technol. 2024 Nov 5;13(11):2. doi: 10.1167/tvst.13.11.2 (PMC11542501; doi:10.1167/tvst.13.11.2)
Supplement: Supplement 13 [file tvst-13-11-2_s013.docx]

**Supplemental Appendix**

*Data Creation Algorithm*

The following is an in-depth explanation of the preprocessing protocol for creating the input images and ground truths from the unlabeled and annotated OCT scans:

1. First, remove the borders and external artifacts of the screenshot and retain only the OCT scan. To do this, a column-wise sum of the image is taken (so if the image is a 2-dimensional array with shape 600 x 1500, the column-wise sum returns an array of 1500 sums). Since the OCT scan portion of the image has the lowest sums (due to its high number of black pixels) the OCT scan portions are extracted by then finding the indexes of the sum array which have the greatest positive and negative difference between the sum at the given index and the sum at the index prior. These two indexes then become the crop bounds for the columns. A constant crop bound is used for the rows because the OCT positions of the scan are mostly in the same spot when looking at the rows.
2. Then, a 512 x 512 crop is to be taken from these extracted screen clippings of the scans which focuses on the actual fovea. To do this, the center of the scan is found. To find the y-coordinate of the center of the scan, a similar method to before is used (using the row-wise sums). To find the x-coordinate of the center, the 80th percentile of pixels on the y-coordinate row are found and their mean is used to calculate the x-coordinate of the center.

Both the ground truth and input images go through these 2 steps. These next 2 steps are done for the ground truths only:

1. The automatically generated Heidelberg Auto segmentations are manually corrected by a clinician as needed. If no corrections are needed, the Heidelberg Auto segmentation lines serve as ground truth.
2. To create a segmentation mask for the deep learning models, the Heidelberg Auto segmentation lines are extracted and the spaces between the layers are labeled by the corresponding layer number. This is done by an algorithm that iteratively scans the columns of the labeled scan, and all pixels contained in the areas between each layer are assigned the class of the layer that precedes them (the pixels between layer 1 and layer 2 were assigned class 1, and so on). The pixels between broken layers are labeled as collapsed layers.

This process is repeated 2 - 3 times for each image depending on the initial images’ OCT scan size by taking crops from the left, center, and right of the image. In total, 1661 pairs of input images and ground truths (each of dimension 512 x 512) were extracted from 572 individual OCT scans collected from 142 patients. The scans are split 80:10:10 at the patient level to create a training, validation, and test set. Figure S1 shows this visually. There was no overlapping between any of the crops.

*Cross Pseudo Supervision architecture information*

The protocol for forward and backpropagation is as follows:

For DeepLabV3 w/ CPS, Regardless of whether the input is labeled or unlabeled, the forward propagation process is the same for both component models. Both component models’ encoding layers consist of a Residual Network (ResNet)[^1^](https://paperpile.com/c/Wd2ku5/XjkrY). Decoding is done through DeepLabV3 using atrous spatial pyramid pooling convolutions (ASPP convolutions)[^2^](https://paperpile.com/c/Wd2ku5/Ha9u6) and a pooling layer. The output of the pooling layer and ASPP convolution are concatenated, then passed through another 1x1 convolution to get the output segmentation.

In the case of Standard UNet w/ CPS - 100%, forward propagation is done by passing the image through the UNet.

Over each iteration in training, a batch of labeled and unlabeled images is passed. Once segmentations are created using the forward propagation protocol, the backpropagation protocol is as follows:

- The labeled loss is found using the equation as shown in the main manuscript using the cross-entropy losses computed on both component model’s predictions against their respective ground truths.
- The unlabeled loss is found using the equation as shown above using the cross-entropy loss computed on both component model’s predictions and corresponding pseudo ground truths.
- The labeled and unlabeled losses are summed and then this total cross-pseudo supervision loss is back propagated across the entire model.

The two models cross-supervise each other on both labeled image and ground mask, and unlabeled data and pseudo mask. The cross-supervision enforces regularization on the features learnt by the two competing models. Specifically, if the models’ feature spaces begin to diverge from each other, the cross pseudo-supervision loss penalizes them. The cross-pseudo supervision loss is calculated by taking the sums of the losses of CPS_1_ and CPS_2_ on labeled and unlabeled input data. Together, this cumulative cross-pseudo supervision loss mitigates the risk of overfitting that can occur for either individual model when trained on supervised input images and their ground truth masks. Furthermore, the pair of models enables both component models to learn features on unlabeled data, thereby utilizing both the supervised and unsupervised image datasets. Finally, cross-supervision regularization helps the models learn a more robust feature space, thereby improving performance when encountering new data. At the end of training, CPS_1_ is used for evaluation on the test set.

6.3 Mean Teacher architecture information

DeepLabV3 w/ MT - 100% consisted of a student model and teacher model referred to hereon as MT_1_ and MT_2_ respectively as shown in Figure S3. When optimizing Mean Teacher, the Mean Teacher loss was used. The Mean Teacher loss consists of 2 losses:

- The loss of MT_1_ on a labeled image and ground truth pair
- The loss of MT_1_ on an unsupervised image and pseudo segmentation by MT_2_ pair

The formula for the total Mean Teacher loss is as follows:

$\left[ Eq. \left( S4 \right) \right] L_{labeled}\left( X_{i},Y_{i} \right)=L_{CE}\left( {MT}_{1}\left( X_{i} \right),Y_{i} \right)+L_{MSE}({MT}_{2}\left( X_{i} \right),Y_{i})$

$\left[ Eq. \left( S5 \right) \right] L_{Unlabeled}(X_{i})=L_{MSE}\left( {MT}_{1}\left( X_{i} \right),MT_{2}\left( X_{i} \right) \right)$

$\left[ Eq. \left( S6 \right) \right] L_{Mean Teacher}=L_{Labeled}+L_{Unlabeled}$

Where $L_{labeled}\left( X_{i},Y_{i} \right)$ represents the labeled loss for the MT_1_ on the i-th labeled input and ground truth pair. $L_{CE}\left( {MT}_{1}\left( X_{i} \right),Y_{i} \right)$ represents the Cross-Entropy loss MT_1_’s prediction and the ground truth. $L_{MSE}({MT}_{2}\left( X_{i} \right),Y_{i})$ represents the mean squared error loss between MT_1_ and MT_2_’s prediction (using MT_2_’s prediction as a pseudo-ground truth). $L_{Unlabeled}(X_{i})$ represents the unlabeled loss for MT_1_ on the i-the unlabeled input image. $L_{Mean Teacher}$ is the total loss for MT_1_ and is the sum of the labeled and unlabeled losses for MT_1_. The parameters of MT_2_ were exponential moving averages of the weights of MT_1_.

Because DeepLabV3 w/ MT also uses a DeepLabV3 decoder with a ResNet50 backbone, forward propagation is the same as described above for DeepLabV3 w/ CPS.

Over each iteration in training, a batch of labeled and unlabeled images is passed. Once segmentations are created using the forward propagation protocol, the backpropagation protocol is as follows:

- The labeled loss is found using the equation as shown above by summing the cross-entropy loss computed on the student model’s prediction against the ground truth and the mean squared error loss computed on the student model’s prediction against the teacher’s prediction.
- The unlabeled loss is found using the equation as shown above using the mean squared error loss computed on the student model’s prediction against the teacher’s prediction
- The labeled and unlabeled losses are summed and then this total mean teacher loss is back propagated across the entire student model. The teacher’s weights are updated accordingly as an exponential moving average of the student’s.

MT_1_ learns from the labeled data and MT_2_, which has its weights updated accordingly in step with the MT_1_. This training framework enables both component models to regulate the other. Mean Teacher is predicated on the assumption that the average of a model’s weights over an epoch are better feature maps than simply the model weight after the final iteration of training. By leveraging unlabeled data, the Mean Teacher framework helps both component models learn a more robust feature space. At the end of training, MT_2_ is saved.

6.4 DConnNet w/ CPS architecture information

DConnNet w/ CPS consisted of two competing DConnNet models. These models, referred to here on as DNet_1_ and DNet_2_, were initialized and followed the same training protocol described above for DeepLabV3 w/ CPS. However, when updating weights, a novel loss function was employed that combined the CPS loss with the DConn loss. This loss function, referred to here on as DConn-CPS loss, was the sum of 4 different DConn losses:

1. The DConn loss from $DNet_{1}$ and the labeled image
2. The DConn loss from $DNet_{2}$ and the labeled image
3. The DConn loss from $DNet_{1}'s$ pseudo ground truth mask on an unlabeled image being compared against $DNet_{2}'s$ prediction.
4. The loss from $DNet_{1}'s$ pseudo ground truth mask on an unlabeled image being compared against $DNet_{2}'s$ prediction.

The formula for the DConn-CPS loss is given below:

$\left[ Eq. \left( 5 \right) \right] L_{DConn-Labeled}\left( X_{i},Y_{i} \right)=L_{DConn}\left( DNet_{1}\left( X_{i} \right),Y_{i} \right)+L_{DConn}(DNet_{2}\left( X_{i} \right),Y_{i})$

$\left[ Eq. \left( 6 \right) \right] L_{DConn-Unlabeled}(X_{i})=L_{DConn}\left( DNet_{1}\left( X_{i} \right),DNet_{2}\left( X_{i} \right) \right)+L_{DConn}(DNet_{2}\left( X_{i} \right),DNet_{1}\left( X_{i} \right))$

$\left[ Eq. \left( 7 \right) \right] L_{DConn-CPS}=L_{DConn-Labeled}+L_{DConn-Unlabeled}$

Where $L_{DConn}$ is the DConn loss as originally defined in [3]. $L_{DConn-Labeled}$ is the sum of the DConn losses of $DNet_{1}$ and $DNet_{2}$ on the ith pair of labeled input and ground truth images. $L_{DConn-Unlabeled}(X_{i})$ is the sum of the DConnNet losses of both component models’ predictions using their competing model’s output as a ground truth label. $DNet_{1}\left( X_{i} \right)$ and $DNet_{2}\left( X_{i} \right)$ represent the respective predictions of $DNet_{1}$ and $DNet_{2}$ on the i^th^ unlabeled input. The total DConn-CPS loss is given by $L_{DConn-CPS}$ as the sum of $L_{DConn-Labeled}$ and $L_{DConn-Unlabeled}$. Once trained, $DNet_{1}$ is used for prediction. Ablation was done with varying amounts of unlabeled data (100% and 50%).

*Baseline Supervised Deep Learning Models*

The Standard UNet has 5 downsampling layers which are followed by a bottleneck layer and then 5 upsampling layers. The model’s output is compared against the ground truth and its cross-entropy loss is back propagated over the model to optimize using the Adam optimizer. Cross-entropy is used as the loss function and the model is optimized using Adam with an initial setting where the learning rate is 0.0003, a beta_1_ of was 0.9, a beta_2_ was of 0.999, and epsilon was as 1e-08. The learning rate is halved every 6 epochs until model performance plateaus. Class weighting is used when calculating the loss so that more emphasis is placed on the thinner layers such as the photoreceptor and collapsed layers. The class weighting for each layer is as follows (class weighting was done during training and during training, the models generate 13-layer outputs, so there are 13 weights rather than 14):

Internal Limiting Membrane (ILM) 3

Retinal Nerve Fiber layer (RNFL) 3

Ganglion Cell layer (GCL) 3

Inner plexiform layer (IPL) 3

Inner nuclear layer (INL) 3

Outer plexiform layer (OPL) 3

External Limiting Membrane (ELM) 9

Photoreceptor 1 (PR1) 9

Photoreceptor 2 (PR2) 9

Retinal Pigment Epithelium (RPE) 9

Collapsed layers 9

Pre-retinal space (Vitreous) 1

Background below retina (Choroid/Sclera) 1

Standard UNet w/ pseudo masks has the same architecture as the Standard UNet and uses the same loss and optimizer as described above.

The second of the baseline supervised models, the Double UNet (Figure S2), consists of two chained UNets, each with 5 downsampling layers, a bottleneck layer, 5 upsampling layers, and skip connections concatenating the output of each downsampling layer with the input of the corresponding upsampling layer. To generate a prediction, the input image is first passed through the first chained UNet, which produces an interim prediction. This interim prediction is concatenated with the initial input image as the input to the second chained, UNet, which generates an output. After the Standard UNet model had been trained, its weights were copied to the first UNet in the Double UNet and frozen. As a result, during the training of the Double UNet, its output is compared against the ground truth, but the loss is only back propagated through the second chained UNet. Cross-entropy loss along with the same class weighting applied when training the Standard UNet was used. Similarly, Adam was chosen as the optimizing algorithm.

*Post-processing*

After each of the different models had generated their respective segmentation output consisting of 13 layers, a post-processing step was done to add a 14th layer to the output before evaluating these predictions against the ground truths of the test set.

All pixels in the prediction that had been labeled as collapsed layers whose corresponding pixel in the input image was also below the 30th percentile of pixel intensity in the input image were labeled as cysts.

**References:**

1. [He, K., Zhang, X., Ren, S. & Sun, J. Deep Residual Learning for Image Recognition. in *IEEE Conference on Computer Vision and Pattern Recognition (CVPR)* vol. abs/1512.03385 770–778 (IEEE, 2015).](http://paperpile.com/b/Wd2ku5/XjkrY)

2. [Chen, L.-C., Papandreou, G., Kokkinos, I., Murphy, K. & Yuille, A. L. Deeplab: Semantic image segmentation with deep convolutional nets, atrous convolution, and fully connected crfs. *IEEE Trans. Pattern Anal. Mach. Intell.* **40**, 834–848 (2017).](http://paperpile.com/b/Wd2ku5/Ha9u6)

3. [Chen, X., Yuan, Y., Zeng, G. & Wang, J. Semi-supervised semantic segmentation with cross pseudo supervision. *Proc. IEEE Comput. Soc. Conf. Comput. Vis. Pattern Recognit.* 2613–2622 (2021).](http://paperpile.com/b/Wd2ku5/lDtO4)
